# A Patient-Derived Xenograft Model of Parameningeal Embryonal Rhabdomyosarcoma for Preclinical Studies

**DOI:** 10.1155/2015/826124

**Published:** 2015-11-30

**Authors:** Jody E. Hooper, Emma L. Cantor, Macgregor S. Ehlen, Avirup Banerjee, Suman Malempati, Peter Stenzel, Randy L. Woltjer, Regina Gandour-Edwards, Neal C. Goodwin, Yan Yang, Pali Kaur, Carol J. Bult, Susan D. Airhart, Charles Keller

**Affiliations:** ^1^Department of Pathology, Johns Hopkins Medicine, 600 N. Wolfe Street, Pathology B-106, Baltimore, MD 21287, USA; ^2^Pediatric Cancer Biology Program, Papé Family Pediatric Research Institute, Oregon Health & Science University, 3181 S.W. Sam Jackson Park Road, Portland, OR 97239, USA; ^3^Department of Pediatrics, Oregon Health & Science University, 3181 S.W. Sam Jackson Park Road, Portland, OR 97239, USA; ^4^Department of Pathology, Oregon Health & Science University, 3181 S.W. Sam Jackson Park Road, Portland, OR 97239, USA; ^5^University of California Davis School of Medicine and Cancer Center, Sacramento, CA 95817, USA; ^6^The Jackson Laboratory, 1650 Santa Ana Avenue, Sacramento, CA 95838, USA; ^7^Champions Oncology, Hackensack, NJ 07601, USA; ^8^Leo Universal, Inc., Torrance, CA 90505, USA; ^9^Children's Cancer Therapy Development Institute, Beaverton, OR 97005, USA

## Abstract

Embryonal rhabdomyosarcoma (eRMS) is one of the most common soft tissue sarcomas in children and adolescents. Parameningeal eRMS is a variant that is often more difficult to treat than eRMS occurring at other sites. A 14-year-old female with persistent headaches and rapid weight loss was diagnosed with parameningeal eRMS. She progressed and died despite chemotherapy with vincristine, actinomycin-D, and cyclophosphamide plus 50.4 Gy radiation therapy to the primary tumor site. Tumor specimens were acquired by rapid autopsy and tumor tissue was transplanted into immunodeficient mice to create a patient-derived xenograft (PDX) animal model. As autopsy specimens had an ALK R1181C mutation, PDX tumor bearing animals were treated with the pan-kinase inhibitor lestaurtinib but demonstrated no decrease in tumor growth, suggesting that single agent kinase inhibitor therapy may be insufficient in similar cases. This unique parameningeal eRMS PDX model is publicly available for preclinical study.

## 1. Introduction

Rhabdomyosarcomas (RMS) are mesenchymal tumors showing skeletal muscle differentiation and represent the most common pediatric soft tissue sarcomas [1]. RMS are subdivided into alveolar (aRMS) and embryonal (eRMS) types, with each category showing distinct histologic appearance, behavior, and response to treatment. Approximately, 60% of RMS are the embryonal type [2]. eRMS presents commonly in the genitourinary and head and neck regions. Compared to aRMS, the average age of onset of embryonal rhabdomyosarcoma is younger, with a majority of cases diagnosed before age 10, and the prognosis is overall more favorable [3]. While the majority of aRMS show balanced translocation with fusion of* PAX3:FOX01* or* PAX7:FOX01*, recent publications suggest that driver mutations (e.g.,* NRAS, KRAS, HRAS, FGFR4, PIK3CA, CTNNB1, FBXW7,* and* BCOR*) may underpin progression in the embryonal subtype of RMS (eRMS) [4, 5]. These studies have suggested that translocation negative aRMS may be more similar to eRMS than the histologic appearance might suggest [6, 7]. eRMS as currently defined overall shows a better prognosis than aRMS with 5-year survival of 80% versus 52% [8].

Besides histologic type, age of onset, and size of tumor, the site of the tumor is highly important for staging and prognosis of RMS [9]. Parameningeal rhabdomyosarcomas (PM-RMS) comprise half of head and neck RMS cases, which also include tumors located in the paranasal sinuses, nasal cavity, middle ear, and the infratemporal and pterygopalatine fossae [8]. Most PM-RMS are of the embryonal type, whereas those with alveolar features show a worse prognosis [10]. Patients with parameningeal eRMS had a 4-year failure-free survival rate of 68% on the most recent Children's Oncology Group study compared to 74% for similar eRMS with primary tumors at other sites [11]. The poorer prognosis of PM-RMS is mainly due to inaccessibility of the site and difficulty in achieving negative surgical margins [12]. Local recurrence is the most common form of relapse, and poor prognostic features include intracranial spread and meningeal involvement [10].

In genetically engineered mice, extra-axial eRMS and aRMS of the head and neck have been generated expressing the* Pax3:Foxo1* fusion gene and cooperating mutations [13–15]. Adult pleomorphic rhabdomyosarcomas have also been shown to develop spontaneously in aging A/J mice [16] or with expression of oncogenic KRAS [6, 7]. Constitutive activation of the Hedgehog pathway in the adipocyte lineage of mice has been associated with the formation of head and neck tumors resembling eRMS [17]. However, preclinical models of parameningeal eRMS tumors are still lacking. Xenograft models of cancer can reflect the heterogeneity of human tumors and create a stromal and vascular milieu not present in cell lines. Preservation of these relationships can more accurately mimic the behavior of human tumors, particularly in their responses to treatment. While immunocompromised test animals and subcutaneous implantation do not precisely mimic the environment within the human body, it is the nearest approximation available for research.

The rarity of PM-RMS and the clinically inaccessible site have made collection of biopsy tissue for xenografting difficult. Here we present an invaluable PDX model, the first such parameningeal eRMS preclinical model, for exploring the biology and preclinical therapeutic avenues in parameningeal RMS, created from tissue obtained at autopsy.

## 2. Case Presentation

**Figure 1:**
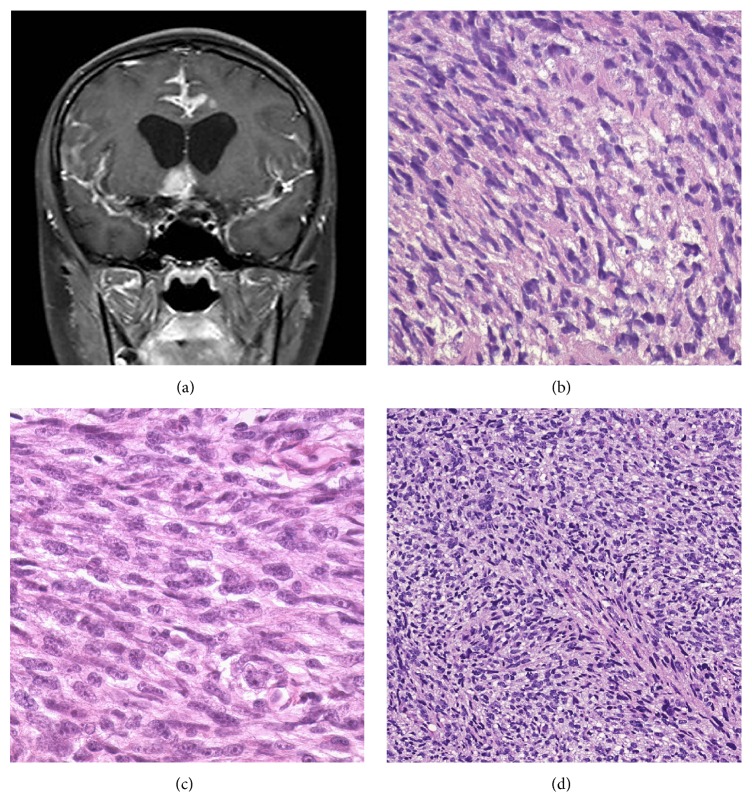
Radiographic imaging and comparative histology of human and PDX tumors: (a) 1 cm × 2 cm enhancing right-sided skull based mass that was invading the hypoglossal nerve canal. (b) H&E slide, 400x of brain biopsy showing sheets of elongated spindled cells with eosinophilic cytoplasm and a myxoid background, consistent with embryonal rhabdomyosarcoma. An H&E slide, 400x from the frontal lobe at autopsy (c), and a section of mouse xenograft, 200x (d), show highly similar morphologic features.

A 14-year-old girl presented with a history of several weeks of persistent headache, hoarse voice, and 20 lb weight loss with tongue deviation on exam. A brain MRI scan showed a 1 × 2 cm enhancing right-sided skull based mass that was invading the hypoglossal nerve canal (Figure 1(a)). Biopsy of the mass showed poorly differentiated tumor with round to spindled cells in a myxoid background (Figure 1(b)). An extensive immunohistochemical panel including positive desmin and myogenin stains was consistent with rhabdomyosarcoma and cytogenetic testing was negative for (2; 13) and (1; 13) translocations, which would be more consistent with alveolar rhabdomyosarcoma. No anaplastic features were noted and the tumor was diagnosed as embryonal rhabdomyosarcoma. The tumor was not amenable to complete surgical resection, and thus the patient was diagnosed with IRS Stage 2, Group III eRMS. The patient received standard chemotherapy with vincristine, actinomycin-D, and cyclophosphamide. Local radiation therapy (RT) was initiated immediately for symptomatic treatment and 50.4 Gy RT to the local tumor produced slow improvement in the vocal cord paralysis and resolution of the tongue deviation.

Approximately 6 months after beginning chemotherapy and 4 months after the completion of RT, the patient developed lower extremity weakness, gait disturbance, incontinence, and headaches. Imaging revealed new diffuse leptomeningeal metastases involving the entire brain and spine. A ventriculoperitoneal shunt was placed, and, to address symptoms of lower extremity weakness and incontinence, the patient emergently received 30 Gy palliative RT to her lower thoracic spine. Shortly after completing RT, she developed difficulty in breathing, seizures, and altered mental status. With ongoing respiratory failure and neurologic deterioration, the family and medical team decided to transition the patient to comfort care only. The patient died shortly after extubation and permission to perform a complete autopsy was given by the family. The study was conducted with appropriate approval by the Institutional Review Board.

The autopsy was performed approximately 28 hours after death. Examination revealed numerous fleshy masses ranging from 1 to 6 cm involving the cerebrum (left frontal and occipital cortex and underlying white matter, cingulate gyrus bilaterally, genu of corpus callosum, left basal ganglia, hypothalamus and left thalamus, right hippocampus, and optic chiasm), cerebellum, and brainstem as well as the leptomeninges. Metastatic tumor was harvested sterilely from several brain sites and placed immediately in RPMI solution with sections fixed in 10% formalin for histology from the same anatomic sites. Histology showed poorly differentiated neoplasm with round to elongated spindled cells in a myxoid background, highly similar to those seen in the previous biopsy (Figure 1(c)).

## 3. Materials and Methods

### 3.1. PDX Model Creation

NSG (NOD.Cg-*Prkdcscid IL2rgtm1Wjl*/SzJ) mice were obtained from The Jackson Laboratory. These highly immune deficient mice have no mature T or B lymphocytes or functional natural killer cells and also have decreased cytokine signaling, rendering them excellent subjects for human tissue engraftment. All studies were done with the approval of The Jackson Laboratory Institutional Animal Care and Use Committee. Tumor pieces taken directly from the patient (50–125 mm^3^) were implanted subcutaneously into the rear flanks of recipient female NSG mice using a trocar. Tumors were allowed to grow to approximately 1000 mm^3^ when the tumors were collected and dissected into approximately 50 mm^3^ fragments. The fragments were serially passaged in NSG mice to create cohorts of mice for drug-testing purposes. To maintain models and minimize genetic drift, fragments from the P0 and P1 passages were frozen in 10% DMSO. These fragments are used to generate low passage number cohorts of tumor bearing mice as needed for study. PDX efficacy studies do not go beyond passage 6.

### 3.2. Genomics

*Gene expression analysis* was performed with the human exon 1.0 ST array (Affymetrix, Santa Clara, CA). Only the initial passaged tumor (P0) was characterized for gene expression because the patient sample could not be collected quickly enough to ensure data quality. PDX gene expression microarrays were processed in the R statistical programming environment [18]. First, arrays were loaded and grouped into probe sets with the BrainArray version 17 CDF [19] and Ensembl human gene annotations (annotation version 70 using the human assembly GRCh37). Individual probe intensities were quantile normalized, and log-transformed, but no background correction was performed. Summarized expression intensities were generated with the probe-level model as implemented by the AffyPLM R package [20], fitting a simple model of the logarithmic intensity for each probe as the sum of a sample effect, a probe effect, and a residual term, with the sample effect representing the summarized intensity of the entire transcript/gene. Mouse contamination effects on the arrays were assessed by hybridization of NSG mouse skin samples on triplicate arrays for the HuGene-1.0-st arrays.

*Copy number variation* for the patient tumor and the P0 PDX tumor were analyzed with the genome-wide human 6.0 SNP array (Affymetrix). The whole-genome allele-specific copy number profiles, fraction of aberrant cells, and tumor ploidy were estimated using ASCAT 2.2 [21, 22]. The input data for ASCAT was generated from the CEL files using the PennCNV-Affy package [23] which extracts the Log R Ratios (LRR) and B-Allele Frequency (BAF) and performs the GC correction. Ensemble genes (human genome annotation version 70) were then annotated with the segmented copy number of the major (CNVa) and minor (CNVb) alleles. Total copy number was computed by adding the values for the major and minor alleles. A segment was defined as loss of heterozygosity (LOH) if the major allele frequency was greater than 0.5 and the minor allele was less than 0.1.

*TruSeq Amplicon Cancer Panel.* Using the Illumina protocol, mutation hotspots from forty-eight (48) cancer-related genes were amplified and sequenced on the Illumina MiSeq sequencer. The TruSeq data were analyzed using a bioinformatics analysis pipeline developed at Jackson Laboratory. Briefly, sequencing reads generated by the platform were initially assessed for mouse contamination using Xenome v1.0.0 [24]. The human specific reads were further subjected to quality control using* NGSQCtoolkit v2.3* [25] and reads with base quality greater than 30 over 70% of bases were used in downstream analysis. High quality reads were mapped to human genome (Hg19) using* BWA* [26]. The resulting alignment was sorted by coordinates and further converted to binary alignment format by Picard tools (http://picard.sourceforge.net/). Subsequently, the* IndelRealigner* and* BaseRecalibrator* modules in the Genome Analysis tool kit (GATK) were used to preprocess the alignments [27, 28]. The realigned and recalibrated BAM file was used as an input to* GATK-UnifiedGenotyper* and the variant calls were restricted to the target region (Agilent sureSelect v4), soft filtered with read depth less than 140. Finally,* Pindel* [29] was used to identify microdeletions and all variants with allele frequency greater than 5% were reported.

### 3.3. Fluorescence* In Situ* Hybridization (FISH)

FISH was performed with formalin-fixed paraffin embedded (FFPE) tissue sections (4 *μ*M, cut from the tissue block PCB-00082PT) using the Standard Operational Procedures (SOP) established at the CLIA-certified Clinical Cytogenetics Laboratory at the Jackson Laboratory for Genomic Medicine. The FFPE slides were baked at 60°C overnight and then deparaffinized with xylene at room temperature for three times, 15 minutes each time. The slides were dehydrated with 100% ethanol at room temperature for 2 minutes and air-dried for approximately 15 minutes. The slides were treated with tissue pretreatment and digestion kits according to the instructions provided by the manufacturer (CytoCell). After digestion, the slides were dehydrated with 70%, 90%, and 100% ethanol, respectively, at room temperature for 2 minutes each, followed by air drying. For denaturation and hybridization, the slides were placed face-down onto a clean H&E slide and the unstained tissue section on paraffin slide was aligned with the corresponding tumor tissue area on the H&E slide. Using a diamond pen, the target tumor area was marked on the slide to be processed for FISH. 10 *μ*L of PAX3 Breakapart probes was applied (Cytocell, LPS 012) and of PAX7 Breakapart probes (Cytocell, LPS 013) onto two separate marked areas, respectively, and the area was covered with a 22 × 22 mm^2^ glass cover slip. The area was sealed with rubber cement and the probes and slide were codenatured on the Hybrite at 94°C for 3 minutes. The slide was placed in a humidified chamber and incubated at the 37°C incubator for 48 hours. For posthybridization wash, the slides were immersed in 0.5X SSC at 72°C for 5 minutes. They were washed 3 times in 1XPBS with 0.025% Tween 20 at room temperature, 2 minutes each time. 10 *μ*L of DAPI was applied to the marked area and it was covered with a 22 × 22 mm^2^ glass cover slip. The edges of the glass cover slip were sealed with nail polish to prevent slides from drying. In this study, we also performed interphase FISH with a normal human control cell line (GM12878) as a control test. The FISH slides were analyzed using the Leica GSL120 image scanner system.

### 3.4. Efficacy Evaluation in Tumor Bearing Mice

Because the patient's tumor sample harbored an ALK mutation, the potent, FDA-approved kinase inhibitor lestaurtinib was chosen for preclinical studies. NSG mice with PDX tumors at passage #2 and an average size of 250~300 mm^3^ were randomized into vehicle control and the lestaurtinib (LC Laboratories, Woburn, MA) treatment groups. Lestaurtinib was prepared in 40% polyethylene glycol 100 (Spectrum New Brunswick, NJ), 10% povidone (ISP), and 2% benzyl alcohol (Spectrum,) as previously described [30]. A total of nine doses were given subcutaneously every 2 to 3 days over a 3-week dosing period at 10 mg/kg. Tumor volumes were measured with a digital caliper 3 times weekly and calculated using the formula: 0.5 × length × width^2^ (mm^3^). Bodyweight, hair coat, and activity were monitored 3 times a week and animal welfare was checked daily. Animals were euthanized when tumors reached a 2,000 mm^3^ tumor endpoint. The experiment was carried out twice. For the first experiment, the vehicle group enrolled 7 mice and the lestaurtinib group 6 mice. The second experiment had 6 mice in both groups.

### 3.5. Statistical Analysis

Tumor volumes with standard error were plotted. The curves were truncated when the number of animals currently on study in particular cohorts decreased to below 50% of the starting animal number for that cohort. The tumor growth delay (TGD) method was used to analyze drug treatment effects on time to tumor endpoint (TTE) for the therapeutic dosing regimen [11]. Statistical significance for median TTE values for treatment comparisons was determined by the Log-rank test with a 95% confidence value for two-tailed statistical analyses.

## 4. Results and Discussion

Five tumor fragments directly from the patient (50–125 mm^3^) were implanted into 5 recipient NSG mice to develop patient derived xenograft models. Within 4 months of tumor implantation, all five mice developed tumors with approximately 1000 mm^3^ volume. Histology was analyzed on all xenografts and showed highly similar features to both the patient's prior premortem biopsy and autopsy specimens (Figure 1(d)). Ki-67 immunohistochemical staining was performed to confirm that human cells were proliferating within xenograft samples. This model and associated data are publicly available and represented in the JAX tumor model repository as PDX model TM00360 [31].

Copy number variation was only assessed for the patient tumor as the P0 PDX tumor failed quality control. Several regions of chromosomal amplification and deletion were observed consistent with previous analyses of rhabdomyosarcomas including gains in chromosomes 2, 8, 11, 12, and 20 [32]. Additionally, gains in chromosomes 5 and 19 were observed. Losses were observed for chromosomes 2, 9, 10, and 11. Genes in the amplified regions included several frequently amplified in soft tissue tumors: CDK4, MDM2, GLI4, and MYC [33].

TruSeq cancer panel targeted amplicon sequencing in both patient and P0 PDX tumor samples revealed an ALK R1181C mutation and no other mutations in the 48 gene panel including the* NRAS*,* KRAS*,* HRAS*,* PIK3CA*,* CTNNB1*, and* FBXW7* genes. The* BCOR* and* FGFR4* genes were not analyzed as they are not included in the TruSeq cancer panel. FISH assay revealed no break-apart involving PAX3 or PAX7.

**Figure 2:**
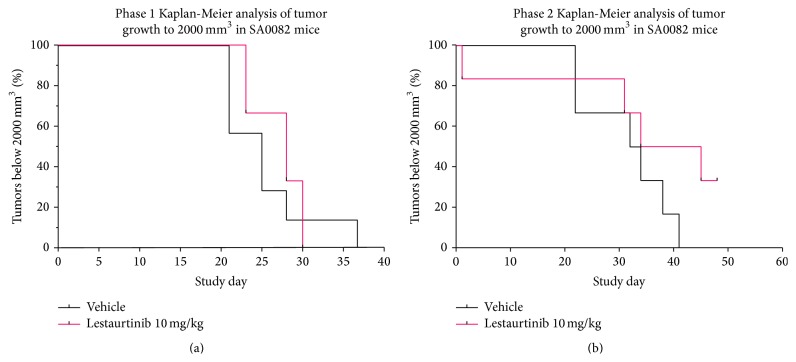
Preclinical testing of lestaurtinib in a parameningeal PDX. (a, b) Kaplan-Meier analysis of PDX mice from two different treatment cohorts.

All mice equally tolerated the treatments with body weights and clinical observations remaining stable for both the vehicle and lestaurtinib treated mice. Tumors grew progressively for both treatments as indicated by mean tumor volumes over time. There was no statistical significance in tumor growth between vehicle and lestaurtinib treatment groups and the Kaplan-Meier survival curves did not differ significantly between vehicle and lestaurtinib treatment groups (*p* > 0.05; Figure 2).

Chemotherapy is a mainstay of treatment for rhabdomyosarcoma, particularly in cases such as our patient when surgical resection is not feasible [34]. Monsma et al. previously utilized a PDX model of alveolar rhabdomyosarcoma, first identifying potential therapies by gene expression profiling and then testing the efficacy of agents* in vivo* [35]. This approach is similar to the one taken in our study. Prior mouse models of eRMS have shown varied pathways to tumorigenesis with individual pathways correlated to specific sites, suggesting that generation of site specific xenografts could lend insight into treatment of rare variants such as PM-RMS [36].

## 5. Conclusions

To our knowledge, this is the first patient-derived xenograft for parameningeal rhabdomyosarcoma. We and others have previously described the importance of research autopsy [37, 38]. This patient succumbed to fulminant eRMS with mass effect after completing radiation and while still receiving multiagent chemotherapy. The ability to obtain treatment resistant eRMS tumor tissue at autopsy and generate PDX is critical to our ability to understand the mechanisms of resistance and to evaluate the impact of novel therapies directed at identified targets. It is of particular interest that while this tumor did demonstrate an* ALK* R1181C mutation, lestaurtinib (which has a 71 nM Kd for ALK) still had no effect [39]. It is possible that this mutation may not drive response to therapy or that most recurrent cancers will not respond to single agent treatment. While our study did not demonstrate response of the xenograft tumors to a pan-kinase inhibitor, generation of the PDX from autopsy tissue provides a tool for identification of potential novel targeted therapies, as well as treatments which might target lestaurtinib resistant tumors in humans.
